# Zeolitic Imidazole Framework/Silica Nanocomposite for Targeted Cancer Therapeutics: Comparative Study of Chemo-Drug Cisplatin (CPt) and Green Platinum (GPt) Efficacy

**DOI:** 10.3390/ijms25063157

**Published:** 2024-03-09

**Authors:** Hend Ghnaim Alotaibi, Eman Al-Abbad, Dana Almohazey, Vijaya Ravinayagam, Sultan Akhtar, Hatim Dafalla, B. Rabindran Jermy

**Affiliations:** 1 Renewable Energy Unit, Basic & Applied Scientific Research Center (BASRC), Imam Abdulrahman Bin Faisal University, Dammam 31441, Saudi Arabia; 2220700032@iau.edu.sa (H.G.A.); ealabbad@iau.edu.sa (E.A.-A.); 2 Department of Chemistry, Collage of Science, Imam Muhammad Bin Saud Islamic University, Riyadh 31441, Saudi Arabia; 3 Department of Nanomedicine Research, Institute for Research and Medical Consultations, Imam Abdulrahman Bin Faisal University, Dammam 31441, Saudi Arabia; 4 Department of Chemistry, College of Science, Imam Abdulrahman Bin Faisal University, Dammam 31441, Saudi Arabia; 5 Department of Stem Cell Research, Institute for Research and Medical Consultations, Imam Abdulrahman Bin Faisal University, Dammam 31441, Saudi Arabia; daaalmohazey@iau.edu.sa; 6 Deanship of Scientific Research & Department of Nanomedicine Research, Institute for Research and Medical Consultations, Imam Abdulrahman Bin Faisal University, Dammam 31441, Saudi Arabia; vrnayagam@iau.edu.sa; 7 Department of Biophysics Research, Institute for Research and Medical Consultations, Imam Abdulrahman Bin Faisal University, Dammam 31441, Saudi Arabia; suakhtar@iau.edu.sa; 8 College of Engineering Research (CER), King Fahd University of Petroleum and Minerals, Dhahran 31261, Saudi Arabia; dmhatim@kfupm.edu.sa

**Keywords:** green synthesis, neem, green Pt, cisplatin, ZIF-8, MOFs, drug delivery, targeted cancer therapeutics

## Abstract

A chemo-drug such as cisplatin is effective for cancer treatment but remains non-specific, is susceptible to drug resistance, and induces several side effects on organ systems. Zeolitic imidazolate framework-8, a type of MOF, has gained attention, including as a drug delivery method for targeted cancer therapeutics. In this study, ZIF-8/Silica nanocomposite was synthesized using a one-pot hydrothermal technique using the Stober technique. We studied the effect of phyto-synthesized GPt and chemo-drug cisplatin CPt on ZIF-8/Silica for targeted efficacy of cancer therapy. The texture, morphology, and chemical environment of Pt on ZIF-8/Silica were analyzed using different characterization techniques such as XRD, FT-IR, BET, diffuse reflectance spectroscopy, SEM-EDX, TEM, zeta potential, and TGA analysis. The isothermal behavior of CPt and GPt adsorption was investigated using isotherm models like Langmuir, Freundlich, and Temkin isotherm. The adsorption kinetics indicating the adsorption efficiency of GPt and CPt are influenced by the concentration of Pt complex and the adsorption sites of ZIF-8/Silica. A high entrapment efficiency and loading capacity of GPt (86% and 4.3%) and CPt (91% and 4.5%) were evident on ZIF-8/Silica. The nanocomposite showed a pH-sensitive Pt release using a dialysis membrane technique. For instance, a high release of GPt (93%) was observed under pH = 6.6 in 72 h, while the release reduced to 50% at pH 7.4 in 72 h. The anti-cancer activity of nanoformulations was studied in vitro using MCF7 (breast cancer cells) and HFF-1 (human foreskin fibroblast) cells. The findings demonstrated that GPt is as effective as CPt; the EC50 value for MCF7 cells treated with ZIF-8/Silica/Cp/PEG was 94.86 µg/mL, whereas for ZIF-8/Silica/GPt/PEG it was 60.19 µg/mL.

## 1. Introduction

Cancer is one of the world’s most feared diseases and is predicted to rise in incidence and mortality by 1% each year until 2030 [1]. The disease is caused by uncontrolled cell division that spreads to surrounding tissue, leading to death. Cells have a specific function, but genes that are normally found in healthy cells can change and become abnormal (genetic mutation), leading to cancer [2,3]. According to the World Health Organization (WHO), cancer caused nearly 10 million deaths worldwide in 2020, or nearly one in six deaths [4]. According to estimations, Arab nations were home to 463,675 new cancer cases, or 2.4% of the world’s incidence, with high malignancy ratios in woman (52.9%). The world female cancer population rate was 186/100,000, whereas the age-standardized incidence rate for Arab women was 137.7/100,000 [5]. Female breast cancer (BC) is the sixth most common cause of mortality in the world (685,000 deaths) (www.who.int/news-room/fact-sheets/detail/breast-cancer), as accessed on 7 March 2024. In 2023, it is anticipated that there will be 43,700 breast cancer-related fatalities in the US (43,170 women and 530 men). According to estimates, 684,996 women worldwide passed away from breast cancer in 2020 [6].

Chemo-drugs such tamoxifen, cisplatin, and anthracyclines (doxorubicin and cyclophosphamide) have been used for the treatment of BC [7]. Cisplatin is widely used in various malignancies such as ovarian cancer, testicular cancer, and solid tumors in the head and neck [8,9]. Johnstone et al. have stated that 50% of patients are treated with platinum compounds [10]. Monotherapy involving cisplatin was found to be effective against triple-negative breast cancer [11]. Nevertheless, the drugs are non-specific and induce neuro-, nephro-, and cytotoxicity to normal cells [12]. Also, low bioavailability and low enhanced permeability and retention effects reduce treatment efficacy [13]. To reduce the toxic effect of cisplatin, several Pt complexes including carboplatin and oxaliplatin have been reported. Several Pt-based complexes are being tested in clinical studies [14]. However, such Pt complexes are either toxic or do not match the drug effects of cisplatin [15]. Synthesis of PtNPs using the green method has attracted great attention due to the lower toxicity of the metallic Pt form and an environmentally friendly synthesis technique [16]. The yield and morphology of nanoparticle formation tend to depend on the plant extract concentrations [17].

Drug repurposing technology offers a new pharmacological/therapeutic route for FDA-approved drugs. Several nanocarriers based on polymeric micelles, porous-structured silica, liposomes, dendrimers, polymers, and mesoporous carbon have been reported for targeted cancer therapeutics [18,19,20,21,22]. In spite of the continuous development of pH-sensitive drug delivery systems, metal–organic frameworks (MOFs) have been interesting for their structural qualities and adsorption properties [23]. MOFs offers high porosity, specific surface area, ease of design, flexibility, and ease of linker modifications for applications in the pharmaceutical industry including adsorption, separation, catalysis, sensing, and drug delivery [24,25,26]. Recently, the zeolite imidazole framework (ZIF-8) has been widely used as a drug carrier for biomedical applications. For instance, the attributes of high porosity, easy modification, structural stability, biocompatible metal ions, and low toxicity make it ideal for controlled drug release systems [27,28,29]. ZIF-8 nanoparticles are effective pH-responsive drug delivery vehicles, but their small pore size results in low drug loading. Mesoporous nanoparticles like SiO_2_, ZnO, and Fe offer high drug loading efficiency and controlled release. Combining ZIF-8 with these nanoparticles offers potential for drug loading, controlled release, and monitoring [30].

In this study, ZIF-8/silica nanocomposite was synthesized using an in situ hydrothermal technique. PtNPs were prepared via the green route and we compared the efficacy with the chemo-drug cisplatin on ZIF-8/Silica through drug adsorption, drug release, and anti-cancer activity. The texture, morphology, and chemical environment of Pt on structured nanocarriers were analyzed using different characterization techniques. The isothermal behavior of drug adsorption was investigated using isotherm models like Langmuir, Freundlich, and Temkin isotherm. The drug release pattern of cisplatin and green Pt was studied using the dialysis membrane technique. The anti-cancer activity of nanoformulations was studied in vitro using MCF7 (breast cancer cells) and HFF-1 (human foreskin fibroblast) cells.

## 2. Results and Discussion

### 2.1. Characterization Studies

#### 2.1.1. UV–Visible Spectra of PtNPs

**Figure 1:**
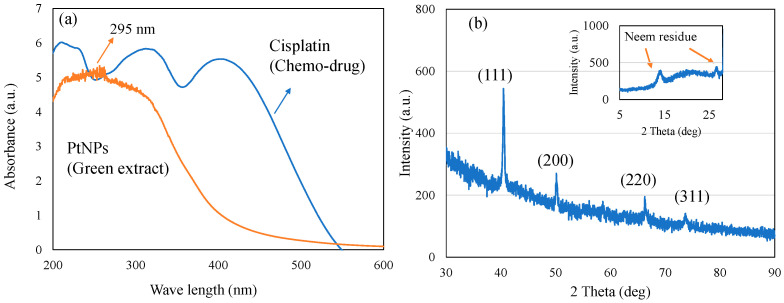
(**a**) UV–visible spectrum and (**b**) X-ray diffraction pattern of green route-synthesized PtNPs and cisplatin.

Green Pt NPs synthesized using neem leaf extract were analyzed using UV–visible spectroscopy (Figure 1a). In the present study, the bioreduction of cisplatin to biogenic Pt^0^ NPs mediated by neem leaves was confirmed with two characteristic changes, including the occurrence of a broad peak between 200 and 400 nm and color change. Pt^4+^ and Cl^−^ ions of plant extract exhibit a sharp absorption peak at about 260 nm due to the process of ligand-to-metal transition. During the reduction process from Pt^4+^ to Pt^0^, a sharp decrease in the absorption peak at 260 nm occurs, and conversely, a wide absorption peak confirms the complete reduction to Pt^0^ species [31]. In our case, the presence of such a broad surface plasmon resonance peak with peak maxima at about 295 nm confirms the effective reduction of cis-[Pt(NH_3_)_2_Cl_2_] and formation of Pt NPs, as reported in earlier literature [32]. In addition, the qualitative confirmation of Pt NPs was also obtained with the sample color changing from yellow to black.

#### 2.1.2. X-ray Diffraction Analysis of Green Pt NPs

Figure 1b shows the X-ray diffraction pattern of PtNPs. The presence of platinum nanoparticles with face-centered cubic crystals was confirmed at 40.5°, 50°, 66°, and 74°, corresponding to the presence of (111), (200), (220), and (311) planes (JCPDS Card 04-0802). A slight shift in the (111) plane from 39.8° towards the higher angle of 40.5° could be attributed to the platinum alloy formation [33]. Further, an effective reduction of cisplatin by neem leaves was also revealed along with the formation of the face-centered cubic crystalline phase of PtNPs. The observed phase diffraction and peaks corresponding to the plant residue match with the earlier literature [34,35].

#### 2.1.3. X-ray Diffraction, FTIR, and BET Surface Area Analysis of Nanocarrier ZIF-8/Silica

**Figure 2:**
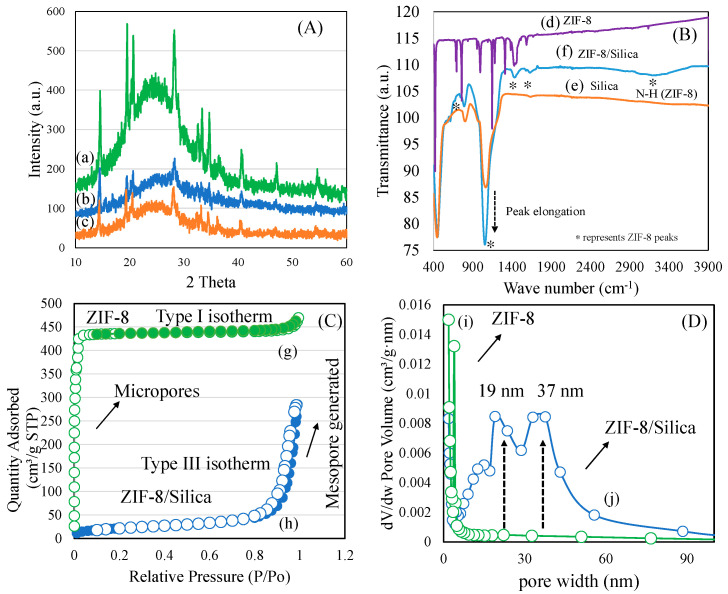
(**A**) X-ray diffraction of (a) ZIF-8/Silica, (b) ZIF-8/Silica/GPt, and (c) ZIF-8/Silica/CPt. (**B**) FTIR spectra of (d) ZIF-8, (e) silica, and (f) ZIF-8/Silica, (**C**) Nitrogen adsorption–desorption isotherm (g) ZIF-8 and (h) ZIF-8/Silica, and (**D**) pore size distribution of (i) ZIF-8 and (j) ZIF-8/Silica nanocomposite.

The phase of ZIF-8 and transformation with silica nanocomposite formation was analyzed using X-ray diffraction analysis (Figure 2A). In the case of parent ZIF-8, typical crystalline peaks were observed at the 2 theta range 5–60° corresponding to the structure of rhombic dodecahedral (sodalite) (Figure S1a). Similarly, cisplatin showed well-resolved crystalline peaks corresponding to the Pt complex (Figure S1b). In the case of ZIF-8/silica nanocomposites (ZIF-8/Silica, ZIF-8/Silica/GPt, and ZIF-8/Silica/CPt), a broad amorphous peak corresponding to the silica appears with peak maxima at 2 theta range of 22° (Figure 2A(a–c)). In addition, residual peaks of ZIF-8 with reduced intensity appear, indicating the integration of ZIF-8 into silica forming a unique ZIF-8/silica nanocomposite formation. Wu et al. (2017) observed a similar peak reduction of ZIF-8 and stated that such ZIF-8/silica transformation occurs due to the decoration process of silica. Further, such integration of silica into ZIF-8 tends to restructure the crystalline structure of ZIF-8 [36]. The functional groups of ZIF-8, ZIF-8/Silica nanocomposite, and silica were analyzed using FTIR spectroscopy (Figure 2B(d–f)). ZIF-8 showed the characteristic stretching vibration bands of imidazole corresponding to -C=N- at 1584 cm^−1^ along with stretching peaks of the five-membered ring between 1311 and 1512 cm^−1^. The stretching and bending of the -C-N bond was observed at 1147 cm^−1^ and 966 cm^−1^, respectively. Further, the -C-H bending of the double bond was observed at 761 cm^−1^, along with ring out-of-plane bending vibration at 695 cm^−1^. The linkage between the Zn^2+^ ion and imidazole ring was confirmed with -Zn-N stretching at 426 cm^−1^. In the case of ZIF-8/Silica nanocomposite formation, the characteristic functional moieties of ZIF-8 confirm the intactness of the sodalite structure, while silica functional peaks indicate the intrusion of silica at the core surface of the ZIF-8. The textural properties of ZIF-8 and ZIF-8/Silica nanocomposite were analyzed using the nitrogen adsorption isotherm technique (Figure 2C,D). ZIF-8 showed the typical micropore filling characteristics at a P/P_0_ value less than 0.2, with a surface area of about 1217 m^2^/g (Figure 2C(g)) (Table 1). The t-plot analysis showed a major portion of the micropore surface area of about 1181 m^2^/g, while the mesoporous surface contributed to about 36 m^2^/g. The pore volume of ZIF-8 was about 0.72 cm^3^/g, with an average pore size distribution of 2.35 nm. In the case of ZIF-8/Silica nanocomposite, the mesoporous silica hybrid formation with ZIF-8 was confirmed with the generation of a hysteresis loop. Interestingly, the micropore surface area reduced proportionately, thereby producing mesopores exhibiting a type III hysteresis loop at a higher P/P_0_ value of 0.9, with a surface area of 75 m^2^/g (Figure 2C(h)). The pore size distribution showed the presence of dual types of mesopores centered at 19 nm and 37 nm, respectively. This shows a unique hybrid nanocomposite formation of silica with ZIF-8 (Figure 2D(i,j)). 

#### 2.1.4. Thermogravimetric Analysis

**Figure 3:**
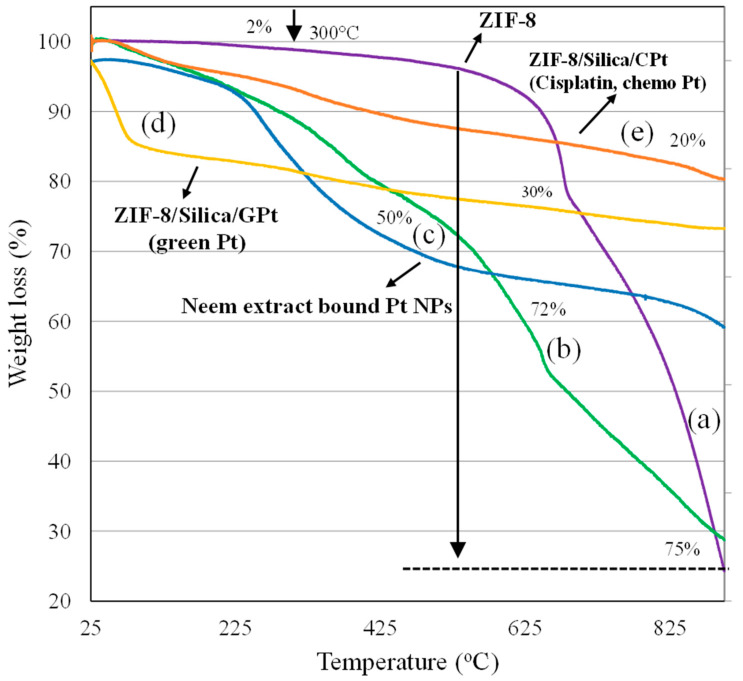
Thermogravimetric analysis of (a) ZIF-8, (b) ZIF-8/Silica, (c) GPt NPs, (d) ZIF-8/Silica/GPt, and (e) ZIF-8/Silica/CPt.

TGA has been widely used to measure the thermal stability and decomposition trend of ZIF-8 and ZIF-8-based nanocomposite. In the present study, the thermal stability and decomposition pathway trend of (a) ZIF-8, (b) ZIF-8/Silica, (c) GPt NPs, (d) ZIF-8/Silica/GPt, and (e) ZIF-8/Silica/CPt were measured using TGA under argon atmosphere (Figure 3). In the case of ZIF-8, an initial weight loss (~2%), attributed to the solvent evaporation and imidazole linker that is physically adsorbed on the external and internal framework of ZIF-8, was observed up to 300 °C [37]. As the temperature increases higher than 300 °C, a distinct weight loss occurs, corresponding to the structural disintegration of ZIF-8 crystals. The decomposition curve subsequently increases steeply, with a total mass loss of about 75% at high temperatures (>300 °C), thereby confirming the carbonization under heat treatment leading to the formation of ZnO. Recently, the presence of core–shell-based ZnO/ZIF-8 nanocomposite exhibited improved thermal stability and moisture endurance ability [38]. Improving the stability of ZIF-8 is critical in physiological conditions. In the present case, the interaction of ZIF-8 with silica through one-pot synthesis was found to improve the thermal stability of nanocomposite ZIF-8/Silica. From the initial temperature to 400 °C, a weight loss of about 19% was observed, indicating the removal of adsorbents from the ZIF-8. Upon further increasing the temperature, a gradual weight loss of about 72% was observed in the ZIF-8/Silica profile, as shown in Figure 3b, which was slightly lower compared to that of ZIF-8 (75%). A variable pattern of weight reduction in the nanocomposite compared to ZIF-8 suggests a slightly improved stability of ZIF-8 nanocrystals with a coating of silica. Figure 3c shows the mass loss of GPt obtained from neem leaf extract. The incurred weight loss between 100 and 310 °C of about 20% was ascribed to cellulosic components, while the decomposition of phytochemicals from neem leaf extract occurs between 300 and 600 °C. The degradation is complete at 600–900 °C, with a total weight loss of about 50%. TGA analysis of GPt-loaded nanoformulation ZIF-8/Silica/GPt incurred a mass loss of 30%, thereby confirming the presence of phytochemicals similar to that of GPt. As expected, the profile of ZIF-8/Silica/CPt comprising cisplatin revealed a lower level of disintegration, with mass loss of 20% between 25 and 900 °C. 

#### 2.1.5. Zeta Potential

**Figure 4:**
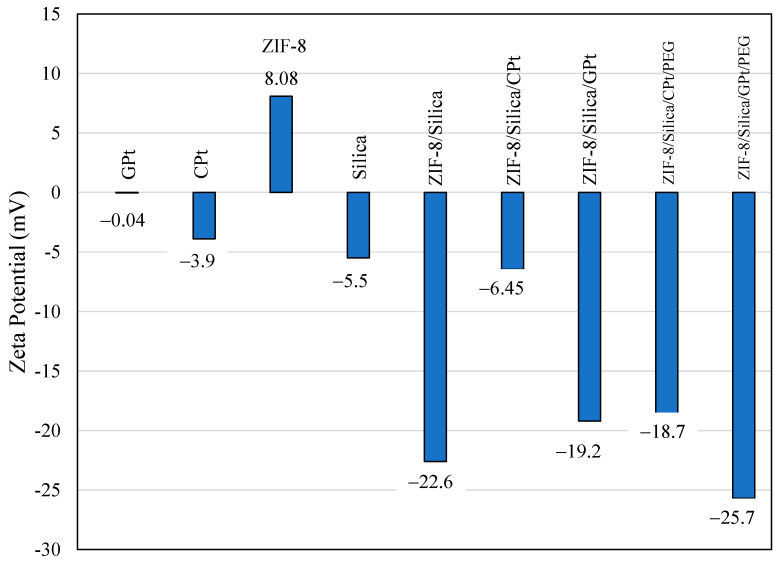
Zeta potential of green PtNPs (GPt), cisplatin (CPt), ZIF-8, silica, ZIF-8/Silica, ZIF-8/Silica/CPt, ZIF-8/Silica/GPt, ZIF-8/Silica/CPt/PEG, and ZIF-8/Silica/GPt/PEG nanocomposites.

The zeta potential measurement of synthesized nanomaterials indicates the charge and colloidal state stability of nanoparticles. Figure 4 shows the zeta potential of green PtNPs (GPt), cisplatin (CPt), ZIF-8, silica, ZIF-8/Silica, ZIF-8/Silica/CPt, ZIF-8/Silica/GPt, ZIF-8/Silica/CPt/PEG, and ZIF-8/Silica/GPt/PEG nanocomposites. As such, the precursor ZIF-8 showed a positive zeta potential of +8.08 mV, while silica showed a negative zeta potential of −5.5 mV. Cisplatin and green-synthesized platinum nanoparticles showed less negative zeta potentials of −3.9 mV and −0.04 mV, respectively. The combination of ZIF-8/Silica showed a large negative value of −22.6 mV, indicating effective composite formation. The observed charge transformation after integration of the positively charged ZIF-8 with negatively charged silica confirms the presence of electrostatic interaction as well as van der Waals interactions [39]. In general, the potential measurements of the samples ranged from −22.6 mV to −25.7 mV, demonstrating dispersion stability caused by repelling interactions between nanoparticles [40]. In the case of CPt functionalization, the zeta potential of ZIF-8/Silica/CPt decreases to −6.45 mV, while ZIF-8/Silica/GPt shows stabilization with green PtNPs, with a value of −19.2 mV, composed of flavonoids and polyphenols. The size distribution measured using dynamic light scattering (DLS) of ZIF-8/Silica shows an average size diameter of 754 nm. After the loading of PtNPs and pegylation, the average particle size decreases to 125 nm. The higher hydrodynamic size distribution observed using the DLS technique compared to TEM is expected and shows the effect of hydration [41].

**Figure 5:**
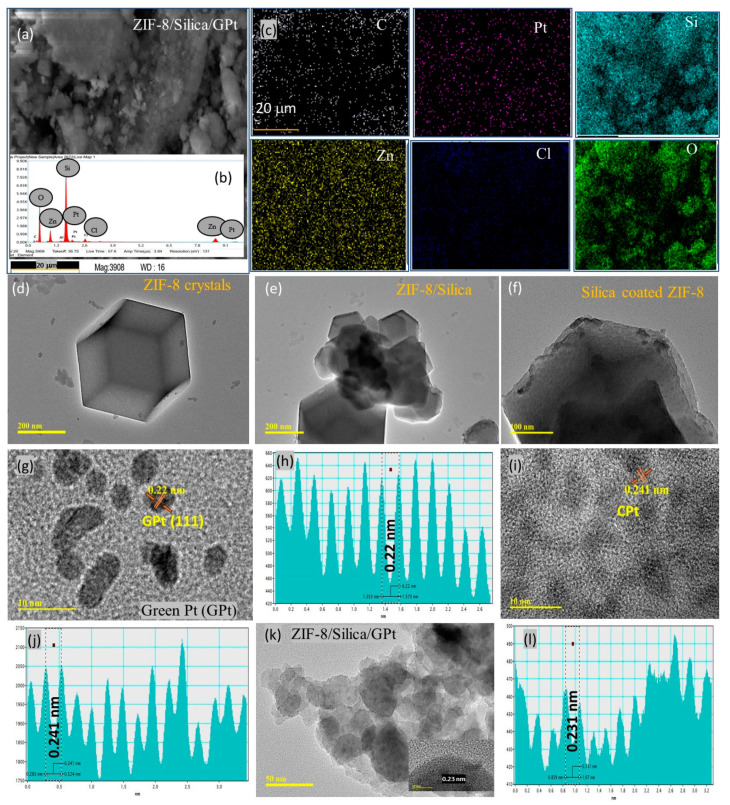
SEM-EDX images of (**a**–**c**) ZIF-8/Silica/GPt, and HRTEM images of (**d**) ZIF-8, (**e**,**f**) ZIF-8/silica, (**g**) green Pt, (**h**) d-spacing measured from lattice fringes of GPt, (**i**) ZIF-8/Silica, (**j**) d-spacing of ZIF-8/Silica/CPt, (**k**) ZIF-8/Silica/GPt, and (**l**) d-spacing of ZIF-8/Silica/GPt.

Figure 5 shows the SEM-EDX images of (a–c) ZIF-8/Silica/GPt, and HRTEM images of (d) ZIF-8, (e and f) ZIF-8/silica, (g) green Pt, (h) d-spacing measured from lattice fringes of GPt, (i) ZIF-8/Silica/CPt, (j) d-spacing of ZIF-8/Silica/CPt, (k) ZIF-8/Silica/GPt, and (l) ZIF-8/Silica/CPt. The utilization of scanning electron microscopy–energy dispersive X-ray analysis (SEM-EDX) enables a rapid and nondestructive assessment of the elemental composition of a given sample, facilitating the prompt identification of its constituent components. The SEM images of ZIF-8/Silica/GPt are shown in Figure 5a. After treatment of ZIF-8 in the Stober synthesis, the formation of ZIF-8/Silica nanocomposite showed a variation in the morphology as agglomerated nanoparticles that are different than the crystal form of ZIF-8. Xu et al. [42] have attributed variation in the ZIF-8 structure to the formation of SiO_2_ shell on the external layers of the ZIF-8 structure, acting as the core. Elemental mapping analysis confirmed the distribution of compositions in ZIF-8/Silica/GPt nanocomposite. In the case of ZIF-8/Silica/GPt, the analysis revealed the abundant presence of carbon, nitrogen, and oxygen, corresponding to the components of neem extract and imidazole of ZIF-8. The crystal mapping reveals a homogeneous dispersion of silica within the ZIF-8/Silica framework (Figure 5a–c). The presence of platinum and chlorine is confirmed on the surface of ZIF-8/Silica/GPt. However, detailed examinations of other characterization techniques, such as UV–visible, X-ray diffraction (Figure 1), and thermogravimetric analysis (Figure 3), clearly showed the different forms of Pt nanoparticles based on the two sources, such as cisplatin alone and neem leaves. 

The morphological features of ZIF-8 (Figure 5d), ZIF-8/Silica (Figure 5e,f), green PtNPs (Figure 5g,h), ZIF-8/Silica/CPt (Figure 5i,j), and ZIF-8/Silica/GPt (Figure 5k,l) were additionally confirmed by the use of high-resolution transmission electron microscopy (HRTEM) investigation. Figure 5d shows that the morphology of raw ZIF-8 exhibits typical, uniform-sized, bigger particle crystallites with rhombic dodecahedral shape in the range of about 500 nm [43]. In the case of ZIF-8/Silica nanocomposite, an interrelated structure consisting of dark-shaped ZIF-8 was observed along with a lighter phase of silica (Figure 5e,f). On comparative scale bar analysis of 200 nm, a significant reduction is confirmed concerning the crystal sizes (250–500 nm) of ZIF-8. Further, the Zn of ZIF-8 in silica was evident with the lattice fringes of Zn^2+^ (0.2515 nm) surrounded by the silica layers. This result was consistent with the results of the XRD diffraction pattern (Figure 2A), FTIR functional groups of ZIF-8 in ZIF-8/Silica (Figure 2B), and elemental composition of SEM-EDX (Figure 5a). The d-spacing arrangement in green PtNPs (Figure 5h), ZIF-8/Silica/CPt (Figure 5j), and ZIF-8/Silica/GPt (Figure 5l) nanocomposites was determined by using lattice fringes. Green platinum nanoparticles (GPt) synthesized using neem leaf extract showed the presence of agglomeration associated with large chunks, typical of the leaf extract-bound nanoparticles (Figure 5g). Green route-synthesized PtNPs using neem leaf extract showed variable particles in the range of 4–10 nm. The presence of metallic Pt (111) in green synthesis was confirmed with the interplanar spacing value of 0.22 nm (Figure 5h). The TEM image of ZIF-8/Silica/CPt shows the dispersion of cisplatin-derived Pt in the range of 4–10 nm on the ZIF-8/Silica support (Figure 5i). In the case of ZIF-8/Silica/CPt, the morphological structure of ZIF-8/Silica after functionalization with cisplatin remains similar to that of parent ZIF-8/Silica nanocomposite. The functionalization of GPt on ZIF-8/Silica showed irregularly shaped particles in the form of clusters attributed to the leaf extract. In the case of GPt loading on ZIF-8/Silica, a highly monodispersed PtNPs occurs with a particle size of about 5 nm, with interplanar spacing values of 0.2 and 0.23 nm (Figure 5k,l).

### 2.2. Effect of Initial Pt Metal Ion Concentration and Isotherm Studies

The initial concentration of Pt metal ions reflects the rate of sorption, which therefore makes it a significant factor to acquire the effectiveness of biosorption. To determine the optimum concentration, metal solution concentrations ranging from 2 to 8 ppm were investigated experimentally for the Pt metal. It is obvious that the removal efficiency (%) was its highest at the maximum concentrations, and vice versa, where the percentage removal (%) was at its lowest at the minimum concentration for both green Pt and cisplatin, where the metal ions were removed as follows for the increased concentration. The removal efficiency (%) of green Pt had a higher magnitude value than cisplatin. 

**Figure 6:**
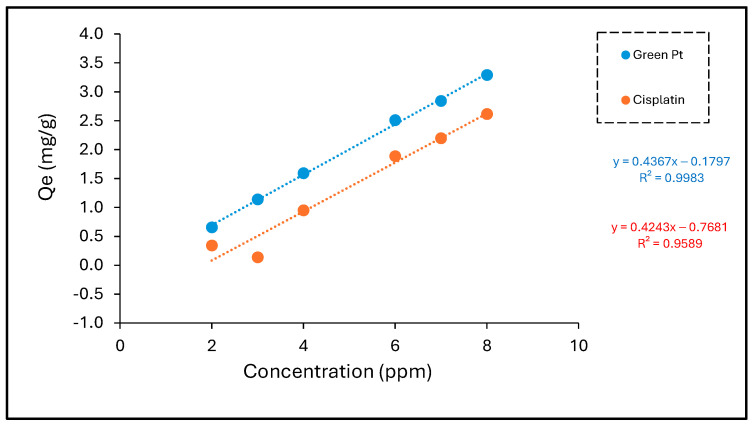
Sorption of green Pt and cisplatin by nanocarrier at different initial concentrations (T = 22 °C, W = 0.1 g, V = 0.05 L, time = 20 h).

As illustrated in Figure 6 and Table 2, the initial solution concentration has a direct relationship with the adsorptive capacity of green Pt and cisplatin onto the nanocarrier. This can be illustrated by the fact that when there is an increase in the concentration of the particles, the metal ions rapidly fill the available sites until they are all occupied. This behavior of ion uptake onto the nanocarrier sample can be explained as a result of more favorable sites for ions to be adsorbed when the metal concentration is increased. In conclusion, it can be estimated that green Pt compounds are more selective for nanocarrier than cisplatin.

### 2.3. Adsorption Isotherm Study

**Figure 7:**
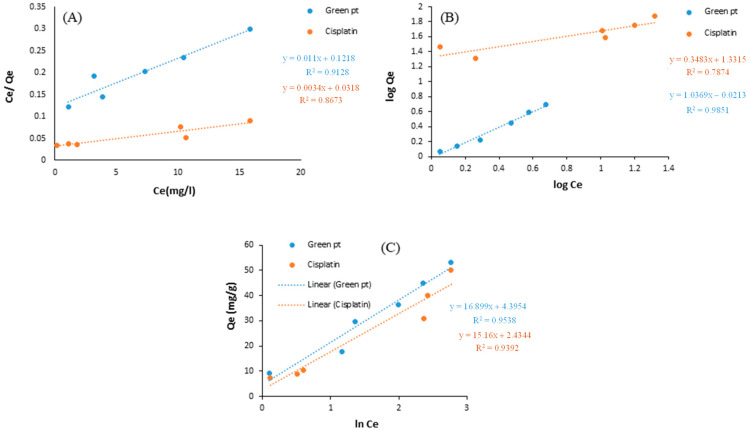
(**A**) Langmuir isotherm, (**B**) Freundlich isotherm, and (**C**) Temkin isotherm of green Pt and cisplatin by nanocarrier.

The adsorption of cisplatin onto the nanocarrier was thus investigated with the help of a linearly established plot of the Temkin, Langmuir, and Freundlich isotherm simulations. The current study demonstrated the distribution method of the adsorbed molecules in the equilibrium state between both the solid and liquid phases, which is referred to as isothermal adsorption. Adsorption isotherms are very helpful in studying the interactions in the adsorption process; they are also useful in optimizing the adsorbent [44]. Figure 7A–C demonstrate the Langmuir, Freundlich, and Temkin isotherm models applied to determine the appropriate isotherm required for green Pt and cisplatin adsorption onto the nanocarrier samples.

#### 2.3.1. Langmuir Isotherm

It is used to describe the adsorption process on a homogeneous surface, consisting of monolayer coverage, without any interactions among the adsorbate (ions). The equation below represents the linear form of the Langmuir isotherms. RL is the separation factor, and the RL value given in the equation reflects the favorability of the adsorption process, as follows:

(1) $$R_{L}=\frac{1}{1+C_{o}Kl}$$

The *R_L_* value ranges between 0 and 1, and depending on the value, the adsorption is considered as follows: 0 < *R_L_* < 1: favorable, *R_L_* = 0: irreversible, *R_L_* = 1: linear, *R_L_* > 1: unfavorable. Figure 7A shows the linear Langmuir isotherm plots for green Pt and cisplatin onto nanocarrier samples. As shown in Table 3, the *R_L_* value was 0.356 for green Pt and 0.318 for cisplatin, indicating the favorability of the adsorption process. Moreover, *K_L_* (Langmuir adsorption constant) represents the affinity of the green Pt and cisplatin samples towards the cations. In the case of green Pt and cisplatin, the values are 0.090 and 0.106, respectively.

The maximum adsorption capacity q_0_ (mg/g) calculated by the Langmuir isotherm is 90.909 mg/g and 294.117 mg/g for green Pt and cisplatin, respectively, onto the nanocarrier sample; the chemical composition with cisplatin obtained a higher cation exchange capacity than that with green Pt.

#### 2.3.2. Freundlich Isotherm

This isotherm equation proposes that as the concentration of the solution increases, the concentration of adsorbate on the surface increases. The assumption for this isotherm model is the heterogeneous adsorptive energies on the surface of the adsorbent. The linear form of the Freundlich isotherm is given in the following equation:

(2) $$q_{e}=K_{F}C_{e}^{\frac{1}{n}}$$

The adsorption parameters of the Freundlich isotherm for green Pt and cisplatin onto the nanocarrier are listed in Table 3. The values of the Freundlich constants were calculated by plotting the Log Ce against *q_e_*, as shown in Figure 7B. The *K_F_* value represents the adsorption ability (capacity), showing a significant difference between green Pt and cisplatin onto the nanocarrier, with values of 2.265 mg/g and 0.081 mg/g for green Pt and cisplatin, respectively. The nanocarrier sample had the highest adsorption capacity, due to its larger surface area, as well as its mesoporous nature. The other Freundlich constant n was in the limit between 1 and 10 and ranged from 0.964 to 2.871, indicating the favorability of adsorption of green Pt and cisplatin onto nanocarrier samples.

#### 2.3.3. Temkin Isotherm

The Temkin isotherm model states that when the adsorbent coverage increases, the adsorption heat of all molecules in the layer decreases linearly; it also considers the indirect effect of adsorbate interactions on the adsorption process. The Temkin isotherm is applicable only at an intermediate range of ion concentrations [45]. Figure 7C shows the linear form of the Temkin isotherm. The linear form of the Temkin isotherm is given in the following equation:

(3) $$q_{e}=\frac{RT}{b}lnAC_{e}$$

Table 3 clearly states that the heat of adsorption (B) using the Temkin isotherm was higher for green Pt than cisplatin. The values using green Pt and cisplatin are 16.899, and 15.160 (J/mol) for the nanocarrier, respectively. Green Pt obtained the highest value of (B), since it positively contributes to the adsorption. The maximum binding energy (A) had the same sequence of selectivity as well. Investigating the previous adsorption isotherms, one can state that the correlation coefficients R^2^ for Langmuir, Freundlich, and Temkin isotherm parameters for green Pt are 0.912, 0.985, and 0.953, respectively, whereas they are 0.867, 0.787, and 0.939 for cisplatin, respectively. The Freundlich adsorption isotherm has been used successfully to model the effects of balance experiments for green Pt, but cisplatin has successfully been modeled with the Temkin adsorption isotherm.

Both the Freundlich and Temkin models were still high and slightly near unity, so that still presents some confidence and provides some evidence that the assumptions of these models could occur simultaneously. Consequently, the Freundlich isotherm and Temkin isotherm fit with the experimental data, assuming a physisorption process on a heterogeneous surface; in addition, the the uptake continued forming multilayers that covered the surface of the samples. From the results, it can be seen that the prepared nanocarrier in the present study displays a good performance for the removal of platinum ions, which might be related to the superior combination advantages of green Pt.

### 2.4. Drug Delivery Study

**Figure 8:**
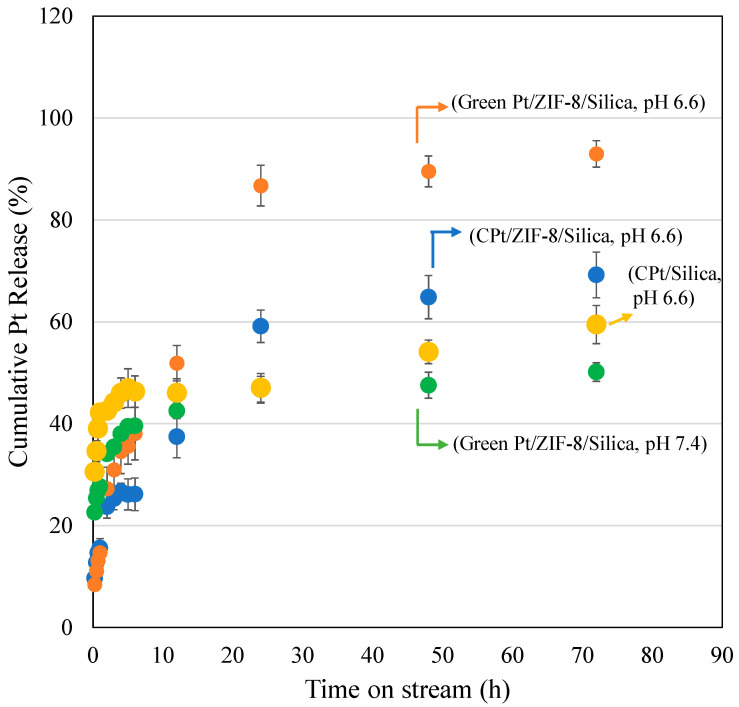
Drug release profile of Pt from cisplatin (ZIF-8/Silica/CPt)-, Silica/CPt-, and green Pt (ZIF-8/Silica/GPt)-functionalized ZIF-8/Silica nanocomposite.

Recently, MOFs have been used as promising drug delivery carriers for several therapeutic chemo-drugs [46]. More interestingly, MOFs exhibited a pH stimulus property for drug delivery of 5-Fluoruracil [47] and doxorubicin [48]. In the present study, the pH stimulus-responsive nature of ZIF-8/Silica/GPt was studied in two pH conditions (pH 6.6 and 7.4) using phosphate-buffered solutions (Figure 8). pH 6.6 mimics the pH of the tumor microenvironment, while pH 7.4 mimics the normal physiological condition. The release of Pt was analyzed using the calibrated curve for PtNPs. In general, the drug release profile shows an initial burst release due to loosely adsorbed drug molecules on the nanocarrier surface, followed by a sustained release. In this study, under the tumor pH of 6.6, a high GPt release of about 93% was observed after 72 h. The release was observed to be higher than cisplatin-bound nanoformulation ZIF-8/Silica/CPt and silica alone, which showed 70% and 60% CPt release after 72 h. In the case of normal physiological pH of 7.4, a slower release of GPt was observed, with a percentage cumulative release of 50% after 72 h. The observed reduced GPt release at normal pH indicates the pH stimulus property of ZIF-8/Silica nanocomposite and the structural stability of ZIF-8. Also, the absence of burst release and the reduced GPt release at pH 7.4 minimize the toxic release of PtNPs that affect the healthy cells. Such high release of the drug at acidic pH conditions is related to the structural decomposition and protonation of the imidazolate linker [49].

### 2.5. In Vitro Cytotoxicity Studies

**Figure 9:**
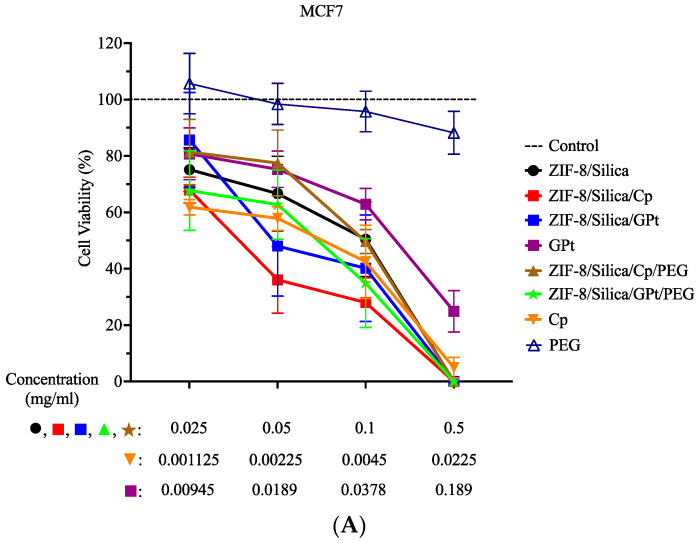

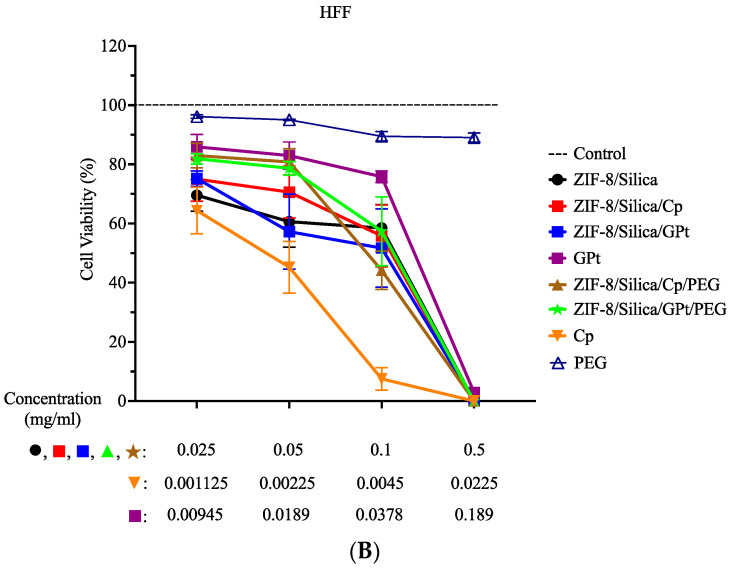
Percentage cell viability. MCF7 (**A**) and HFF (**B**) cells were treated with the following conditions: ZIF-8/Silica, ZIF-8/Silica/Cp, ZIF-8/Silica/GPt, GPt, ZIF-8/Silica/Cp/PEG, ZIF-8/Silica/GPt/PEG, Cp, and PEG for 48 h.

The cell viability assay (MTT) was performed on MCF7 and HFF cells to compare the cytotoxic efficacy of cisplatin- (Cp) versus the green Pt (GPt)-loaded ZIF-8/silica nanocomposite, as shown in Figure 9 and the statistical analysis in Table S1 (Supplementary Figure S2 is the data from Figure 9 presented in Log scale). Cells were treated with the following nanoformulations: ZIF-8/Silica (control), ZIF-8/Silica/Cp, ZIF-8/Silica/GPt, GPt, ZIF-8/Silica/Cp/PEG, and ZIF-8/Silica/GPt/PEG. As mentioned in Section 3.7.1, the treatment concentrations of Cp and GPt leaf extract were adjusted to reflect actual concentrations in the nanocomposite. Thus, cell viability results of the different treatment conditions within the same dose (i.e., first, second, third, and fourth dose) are comparable. In this study, the nanocarrier ZIF-8/Silica resulted in low cytotoxicity at the lower doses (0.025 and 0.05 mg/mL), while it exhibited a cytotoxic effect with further increase in dose on both MCF-7 and HFF. The addition of the chemotherapeutic drug (Cp) and green Pt (GPt), forming treatment groups ZIF-8/Silica/Cp and ZIF-8/Silica/GPt, resulted in a significant cytotoxicity in MCF7 cells in a dose-dependent manner. In contrast, the same nanoformulations showed a lower toxic effect in HFF cells than that seen in MCF7. The different effects on both cell lines were seen in the half-maximal effective concentration (EC50) values (Table 4). EC50 values for ZIF-8/Silica/Cp and ZIF-8/Silica/GPt in MCF7 were 39.72 µg/mL and 62.11 µg/mL, respectively (Table 4), while EC50 values for the same nanoformulations on HFF were 94.01 µg/mL and 75.05 µg/mL, respectively (Table 4). After pegylation, treatment with ZIF-8/Silica/Cp/PEG and ZIF-8/Silica/GPt/PEG resulted in a slightly lower toxic effect compared to treatment with nanoformulations without PEG. This is due to PEG covering the external surface of the nanoformulations, which prevents the direct interaction of Pt with cells. However, increasing the dose to 0.1 and 0.5 mg/mL resulted in a significant reduction in cell viability. Although treatment with the pegylated Cp nanoformulation resulted in similar cell viability % in both cell lines, pegylated GPt nanoformulation resulted in better viability in HFF compared to that in MCF7. In MCF7 cells, ZIF-8/Silica/GPt/PEG resulted in the following cell viability: 67.69%, 62.73%, and 34.92% for 0.025, 0.05, and 0.1 mg/mL, respectively. However, the same conditions in HFF resulted in the following viability: 81.86%, 78.64%, and 57.38% for 0.025, 0.05, and 0.1 mg/mL, respectively. The statistical analysis on MCF7 (A, B) and HFF (C, D) was compared to the “no treatment control” (A, C) and cisplatin (B, D), in which the same corresponding concentrations were used (i.e., the first dose of the nanocomposites was compared to the first dose of cisplatin) (Table S1). The green Pt compound activity was comparable to the chemotherapeutic drug cisplatin, thus providing a significant breakthrough in identifying a new Pt compound that is as active as cisplatin.

The mechanism of action of the different components of the neem extract had been extensively explored in the literature. Studies showed that using neem extract on MCF7 and HeLa cell lines resulted in a reduction in Cyclin D1 and an increase in the Sub-G0 cell cycle phase and the pro-apoptotic protein Bax [50]. Another study found that the neem extract resulted in an induction of caspase-dependent and -independent apoptosis and an activation of the autophagy cascade in colorectal, prostate, and breast cancer cell lines [51]. Neem extract also resulted in inhibition of cell growth and migration accompanied by a reduction in phosphorylated STAT3 and AKT levels in oral cell carcinoma cell lines [52]. On the other hand, in vivo studies showed that the neem extract resulted in a reduction in tumor volume and inflammatory cytokines, which are associated with cancer progression, including NF-kB, COX2, IL-1, IL-6, and TNF [52]. Moreover, neem treatment in rats with chemically induced mammary tumors resulted in inhibition of tumor progression, which lasted even after stopping the treatment [53]. Further examination of these rats revealed that neem treatment resulted in increased levels of p53, Bax, and PTEN and reduced levels of Bcl-2, VEGFA, Cyclin D1, Cdk2, Cdk4, NF-kB, and MAPK1 [53]. In another study, monocytes were isolated from blood samples of cervical cancer patients, which were then treated with neem [54]. High levels of caspase −3, −8, and −9 were detected in the treated monocytes along with decreased levels of secreted TNF and increased secretion of IFN-g [54]. In addition, an in silico analysis was conducted to predict the target proteins of the neem extract components [55,56]. The predicted targets included crucial proteins that are involved in proliferation and cell cycle signaling, such as EGFR, cyclin-dependent kinases, PI3K, Akt, ERK, and GSK-3b. Moreover, some components were predicted to inhibit the estrogen receptor alpha (ERα) protein, thus inhibiting the cell proliferation in prostate and breast cancer [55]. These studies provide a preview of the possible mechanism of action of the neem extract, as it increases the pro-apoptotic genes/proteins and decreases the anti-apoptotic, angiogenic, cell cycle, and pro-survival proteins, thus halting the tumor progression.

Treatment strategy utilizing nanocomposites loaded with chemotherapeutic drugs and biomolecules has shown great potential as a cancer treatment strategy. Studies have shown that combination treatment of nanocomposites loaded with chemotherapeutic drugs and biomolecules with an immunomodulatory drug maximizes the benefit of immunotherapy in cancer treatment [57,58]. The combination treatment significantly improved in vivo anti-tumor efficacy and survival rate, and reduced the tumor volume in mice, suggesting effective tumor suppression, with a boosted anti-tumor immune response at a lower therapeutic dose and a lower risk of side effects.

**Scheme 1:**
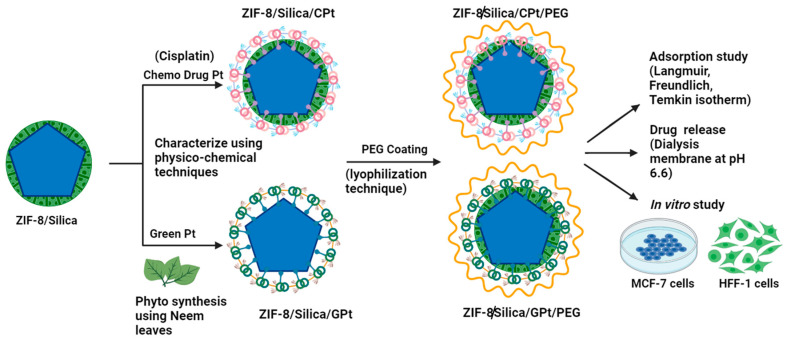
Graphical presentation of ZIF-8/Silica composite formation and green and chemo-drug Pt functionalization, followed by PEG coating for potential targeted cancer therapeutics.

Our group investigated several novel nanocomposite designs to improve the efficacy of cisplatin. We previously showed that MCF7 cells treated with CuFe_2_O_4_/HYPS/cisplatin nanoparticles had an EC50 of 180.89 µg/mL [59]. Moreover, MCF7 cells treated with MnFe_2_O_4_/silica/cisplatin had an EC50 of 48.43 µg/mL, while cells treated with MnFe_2_O_4_/GO/cisplatin had an EC50 of 85.36 µg/mL [60]. In this study, we examined the use of ZIF-8/Silica as a base nanocomposite and compared the effects of the widely used cisplatin versus a green platinum product (GPt) (Scheme 1). Our results showed that MCF7 cells treated with ZIF-8/Silica/Cp/PEG and ZIF-8/Silica/GPt/PEG had an EC50 of 94.86 µg/mL and 60.19 µg/mL, respectively. Although these results might seem higher than our previously reported constructs, we are introducing a new green Pt compound that has comparable efficacy to cisplatin.

## 3. Materials and Methods

ZIF-8 (Basolite Z1200), Tetraethyl orthosilicate (reagent grade, 98%), ammonia (25%, Suprapur), sulfuric acid (ACS reagent, 95–98%), methanol (ACS reagent, 99.8%), ethanol (BioUltra, 99.8%), and polyethylene glycol (Mwt~400) were purchased from Sigma Aldrich, St. Louis, MO, USA. 

### 3.1. PtNP Synthesis

Neem leaves were purchased from the local herbal market. Aqueous extracts of neem leaves were prepared by dissolving 5 g of neem leaf powder in 100 mL of distilled water in an Erlenmeyer flask and boiled at 90 °C for 30 min. Then, the suspension was filtered through Whatman No. 1 filter paper and preserved at 4 °C. Cisplatin solution (1 mM) was prepared and used as a source for platinum, while neem leaf extract acts as a reducing agent. After the preparation of solution mixtures, 20 mL of neem leaf extract solution was added to 180 mL of metal solution dropwise and stirred for 1 h and kept stationary overnight. After the addition of leaf extract, the color changed from green to brown. The sample was recovered by gradually heating the solution from 40 °C to 150 °C for 3 h.

### 3.2. ZIF-8/Silica

The zeolitic imidazolate framework (1 g) was dissolved in 50 mL of deionized water and 50 mL of ethanol mixture and stirred for 10 min. Then silica source TEOS (5 mL) and 12 mL of NH_3_ solution (25%) were added to the ZIF-8 mixture at pH ~9 and stirred for 2 h. The solution was centrifuged, washed twice, and the mixture was placed in the oven at 90 °C and dried overnight. 

### 3.3. ZIF-8/Silica/Pt

The Pt complex (CPt and GPt) (30 mg) was functionalized with ZIF-8/Silica (600 mg), corresponding to a ratio of 0.05, by stirring under normal saline solution. The amount of Pt complex present in the sample was calculated based on the Pt present in the collected filterant solution along with washing (5 mL) using UV–visible spectroscopy. The entrapment efficiency (EE%) and the loading capacity (LC%) of the two formulations (chemo and green Pt) were calculated using the following formula:

(4) EE%=Pt in ZIF−8/Silica_Initial Pt×100

(5) LC%=Initial amount of Pt−supernatant Pt_ZIF−8/Silica×100

The final Pt complex in the nanocarrier was about 27.2 mg. The calculation revealed that ZIF-8/Silica with cisplatin had an EE of 91% and LC of 4.54%, while green Pt showed an EE of 86% with LC of 4.31%. After drug functionalization, both nanoformulations were pegylated using the lyophilization technique. In this step, liquid solution PEG with a molecular weight of 400 (14 μL) was mixed in distilled water (3 mL). Then, 150 mg of the sample (ZIF-8/Silica/CPt or ZIF-8/Silica/GPt) was added and stirred overnight. Then, the sample was lyophilized, recovered, and stored at 4 °C.

### 3.4. Characterization Techniques

The structural transformations of the crystalline phase of ZIF-8 upon nanocomposite formation with silica and green Pt were analyzed and confirmed using the powdered X-ray diffraction technique (Miniflex 600, Rigaku, Japan). ZIF-8 and nanocomposite textural variations were measured using the N_2_ adsorption technique (ASAP-2020 plus, Micromeritics, Norcross, GA, USA). About 20 mg of sample was placed into the sample cell. Prior to surface area analysis, the sample moisture was removed by pretreatment at 50 °C for 2 h under a vacuum. The chemical states of green-synthesized Pt NPs, nanocarrier, and nanoformulations were measured using DRS-UV–visible spectroscopy analysis (JASCO, Tokyo, Japan). The degradation patterns of ZIF-8, ZIF-8/Silica, and nanoformulations were measured using TGA-DTA (STA 6000, Perkin Elmer, USA). The zeta potential of Pt and nanocomposites was analyzed using Zetasizer Nano ZS (Malvern Panalytical Ltd., Worcestershire, UK). About 5 mg of nanoformulation was sonicated (30 min) in 10 mL of PBS buffer solution (pH 7.4). For the analysis, the sample was transferred into a quartz glass cell and measured. The pegylated samples were dried using automated freeze dryer Lyovapor L-200 (Buchi, Labortechnik AG, Flawil, Switzerland). SEM-EDS of the selected sample ZIF-8/Silica/GPt was characterized using JSM-6610LV from JEOL Ltd., Tokyo, Japan. Elemental mapping was obtained by energy dispersive spectroscopy (EDS) using Aztec Version 4.0 software (Oxford, UK). The morphology of ZIF-8, ZIF-8/silica, green Pt, ZIF-8/Silica/CPt, and ZIF-8/Silica/GPt was analyzed using transmission electron microscopy (JEM2100F from JEOL Ltd., Tokyo, Japan). The lattice fringes were measured using Gatan digital micrograph software (version 1.84.1282).

### 3.5. Adsorption Study

The isothermal behavior of drug adsorption was investigated using isotherm models like Langmuir, Freundlich, and Temkin isotherm [31,44]. In the first step, 200 ppm of cisplatin or green platinum Stock Solution (S.S (1)) was prepared by dissolving 50 mg of source in 50 mL of release media (pH 6.6). Then, different ppm solutions (2–8 ppm) were prepared. For adsorption, 10 mL of each ppm solution was added to a conical flask, 100 mg of ZIF-8/Silica was added, and the adsorption study was performed for 20 h in a shaker. The shaking was maintained at 50 rpm. After that, the solution mixture was filtered, and the supernatant was analyzed for platinum concentration using Inductively Coupled Plasma Spectroscopy (ICP).

The formula for adsorption is as follows:

(6) $$Qe=\frac{\left(C_{0}-C_{e}\right)\;V}{W}$$

where

*C_e_* (mg/L): unloaded amount at equilibrium conditions;
*C*_0_ (mg/L): initial concentration;
*V* (L): amount of solution (volume);
*W* (g): weight of adsorbent ZIF-8/Silica.

The formula for the removal capacity Q (mg g^−1^) at varying contact times is as follows:

(7) $$Qt=\frac{\left(C_{0}-C_{e}\right)\;V}{W}$$

### 3.6. Drug Release Study

The release profile of ZIF-8/Silica/CPt/PEG and ZIF-8/Silica/GPt/PEG nanoformulations was studied using the dialysis membrane technique. Prior to the study, the membrane was activated by placing it in normal saline at 37 °C under a magnetic stirrer. After that, 15 mg of sample was dispersed inside the membrane and placed in a 25 mL beaker (pH 6.6). The tumor acidic pH solution of 6.6 was prepared by dissolving each vial of pellets (P8165, Sigma-Aldrich) in 3.8 L of distilled water. In the case of normal pH of 7.4, the pH of the solution was maintained using tablets of phosphate-buffered saline (P4417, Sigma-Aldrich). Periodically, at different time intervals (0.25, 0.5, 0.7, 1, 2, 3, 4, 5, 6, 12, 24, 48, and 72 h), 5 mL of solution was withdrawn, replaced with fresh normal saline, and the contents were analyzed for Pt content using UV–visible spectroscopy.

### 3.7. In Vitro Study

The cytotoxic efficiency of our nanocomposites with green cisplatin was compared to that using nanocomposites prepared with the commercially available cisplatin. The cytotoxicity was conducted using the following cell lines: human mammary adenocarcinoma cell line (MCF7) and the human foreskin fibroblasts (HFFs). Cell lines were purchased from ATCC (American Type Culture Collection), Manassas, Virginia, United States. Cells were cultured in DMEM, 10% HI-FBS, 1% Penicillin Streptomycin, and 1% MEM-NEAA. Cultures were incubated at 37 °C and 5% CO_2_ under humidified conditions. For the cytotoxicity assay, cells were cultured in a 96-well plate at 20,000 cells/well. All cell culture reagents were purchased from Thermo Fisher Scientific, Waltham, MA, USA.

#### 3.7.1. Cell Treatment

Cells were treated for 48 h under the following conditions: ZIF-8/Silica, ZIF-8/Silica/Cp, ZIF-8/Silica/GPt, GPt, ZIF-8/Silica/Cp/PEG, ZIF-8/Silica/GPt/PEG, Cp, and PEG. The treatment concentrations for all groups, except GPt and Cp, were 0.025, 0.05, 0.1, and 0.5 mg/mL.

##### Cell Treatment with Cp

According to the drug loading experiment, there was 0.045 mg of Cp/1 mg of ZIF-8/Silica/Cp and ZIF-8/Silica/Cp/PEG. Thus, the calculated actual concentrations of Cp in the experimental concentrations of the nanocomposites were 0.001125, 0.00225, 0.0045, and 0.0225 mg/mL. For example, 0.5 mg/mL of ZIF-8/Silica/Cp or ZIF-8/Silica/Cp/PEG will actually contain 0.0225 mg/mL of Cp.

##### Cell Treatment with GPt

According to the drug loading experiment performed after leaf extraction, there was 0.1189 of GPt/1 mg of the leaf extract. The leaf extract stock solution was prepared at a concentration of 1.89 mg/mL, which means that the stock actually contains 0.225 mg/mL of GPt. Furthermore, the drug loading experiment performed after nanocomposite preparation showed that there was 0.045 mg of GPt/1 mg of ZIF-8/Silica/GPt and ZIF-8/Silica/GPt/PEG. Thus, the calculated actual concentrations of GPt in the experimental concentrations of the nanocomposites were 0.00945, 0.0189, 0.0378, and 0.189 mg/mL. For example, 0.5 mg/mL of ZIF-8/Silica/GPt or ZIF-8/Silica/GPt/PEG will actually contain 0.0225 mg/mL of GPt. Thus, if 1.89 mg/mL of the leaf extract stock solution contains 0.225 mg/mL of GPt, then 0.0225 mg/mL of GPt in our example is equivalent to a concentration of 0.189 mg/mL of the GPt leaf extract solution.

#### 3.7.2. Cell Viability Assay

The MTT (3-(4,5-dimethylthiazol-2-yl)-2,5-diphenyltetrazolium bromide) assay was used to assess the cytotoxic capability of our Cp-green containing nanocomposite compared to the Cp-chemo containing nanocomposite. The principle of the assay depends on the ability of live cells to convert the yellow-colored MTT to the blue-colored formazan crystals. Briefly, MTT (Sigma-Aldrich) solution was added to cells at a concentration of 0.5 mg/mL and incubated at 37 °C for 4 h [61]. After the incubation period, 0.04 N HCl isopropanol was added to dissolve the formazan crystals. After that, a plate reader SYNERGY-neo2 (BioTek U.S., Winooski, VT, USA) was used to measure the absorbance at 570 nm. Two readings were taken before and after the addition of MTT. The first reading was subtracted from the second reading to eliminate unspecific interference. The analysis was performed in comparison with the untreated control. The percentage viability was calculated as treated/control × 100. These results were then used to calculate the half maximal effective concentration (EC50) using GraphPad Prism 10.0.3 software.

#### 3.7.3. Statistics

The cytotoxicity assay was repeated in three biological replicates. GraphPad Prism 10.0.3 software was used for statistical analysis. The analysis was performed using two-way ANOVA with Dunnett’s multiple-comparison post hoc test; diagrams represent error bars ± standard error of the mean (S.E.M).

## 4. Conclusions

The chemo-drug cisplatin is effective for cancer treatment but also affects the normal cells and exerts neuro-, nephro-, and cytotoxic effects. In this study, green platinum nanoparticles with a face-centered cubic crystalline phase were synthesized using neem leaves, and their therapeutic efficacy was compared with that of cisplatin. Silica-coated ZIF-8 nanocarrier with a mesoporous surface area of 75 m^2^/g, with an average pore size distribution centered at 23.5 nm, was developed using the Stober technique. The isothermal behavior of drug adsorption was investigated using isotherm models like Langmuir, Freundlich, and Temkin isotherm. The correlation coefficients R^2^ for Langmuir, Freundlich, and Temkin isotherm parameters for GPt are 0.912, 0.985, and 0.953, respectively, whereas they are 0.867, 0.787, and 0.939 for CPt, respectively. The Freundlich adsorption isothermal has been used successfully to model the effects of balance experiments for green Pt, but cisplatin’s behavior was successfully modeled by the Temkin adsorption isotherm. The Pt release pattern of cisplatin and green Pt was studied using the dialysis membrane technique. In this study, under the tumor pH of 6.6, a high GPt release of about 93% versus 70% CPt was observed after 72 h. In the case of a normal physiological pH of 7.4, a slower release of GPt (50%) was found after 72 h. The cytotoxic effectiveness of CPt- and GPt-loaded ZIF-8/silica nanocomposite was compared using a cell viability test (MTT) on MCF7 and HFF cells. The findings demonstrated that the EC50 value for MCF7 cells treated with ZIF-8/Silica/Cp/PEG was 94.86 µg/mL, whereas for ZIF-8/Silica/GPt/PEG, it was 60.19 µg/mL. Conclusively, the developed novel green Pt compound is just as effective as cisplatin. The developed green synthesis technology can be advantageous for cancer treatment due to its high bioavailability, low cost, and environmentally beneficial process. Further, the results highlight the requirement of study using in vivo models to gain a deeper insight into the greener Pt-based nanoformulation effect.

## Figures and Tables

**Table 1:** Textural properties of ZIF-8 and ZIF-8/Silica nanocomposite.

Sample	BET Surface Area (m^2^/g)	t-Plot microSA (m^2^/g)	MesoSA (m^2^/g)	Pore Volume (cm^3^/g)	Pore Size (nm)
ZIF-8	1217	1182	35	0.72	2.3
ZIF-8/Silica	75	-	75	0.44	23.5

**Table 2:** Sorption of green Pt and cisplatin by nanocarrier at different initial concentrations.

*C*_0_ (ppm)	Green Pt	Removal Efficiency (%)	Cisplatin	Removal Efficiency (%)
	Qe (mg/g)		Qe (mg/g)	
3	1.14	75.90	0.14	9.00
4	1.59	79.50	0.95	47.50
6	2.51	83.67	1.89	62.83
7	2.84	81.14	2.20	62.71
8	3.29	82.25	2.62	65.38

**Table 3:** Langmuir, Freundlich, and Temkin models parameters for sorption of green Pt and cisplatin.

	Langmuir Model				Freundlich Model			Temkin Model		
	q_0_ (mg/g)	*K_L_* (L/mg)	*R_L_* (L/mg)	R_2_	*K_f_* (mg/g)	n	R^2^	B (J/mol)	A (L/g)	R^2^
Green Pt	90.909	0.090	0.356	0.912	2.265	0.964	0.985	16.899	1.296	0.953
Cisplatin	294.117	0.106	0.318	0.867	0.081	2.871	0.787	15.160	1.174	0.939

**Table 4:** EC50 values (µg/mL) for MCF7 and HFF cell lines.

Treatment Group	EC50 µg/mL	
	MCF7	HFF
ZIF-8/Silica	83.04	82.02
ZIF-8/Silica/Cp	39.72	94.01
ZIF-8/Silica/GPt	62.11	75.05
ZIF-8/Silica/Cp/PEG	94.86	90.06
ZIF-8/Silica/GPt/PEG	60.19	107.6
Cp	2.849	1.965
GPt	31.47	55.09

## Data Availability

All related data can be provided by the corresponding authors upon request.

## Supplementary Materials

The supporting information can be downloaded at: https://www.mdpi.com/article/10.3390/ijms25063157/s1.
