# Supplementary material for: Zeolitic Imidazole Framework/Silica Nanocomposite for Targeted Cancer Therapeutics: Comparative Study of Chemo-Drug Cisplatin (CPt) and Green Platinum (GPt) Efficacy
Source: Int J Mol Sci. 2024 Mar 9;25(6):3157. doi: 10.3390/ijms25063157 (PMC10969832; doi:10.3390/ijms25063157)
Supplement: Supplementary file 1 [file ijms-25-03157-s001.zip › ijms-2829480-supplementary.pdf]

Table S1. Statistical analysis on MCF7 (A, B) and HFF (C, D) as compared to the “no treatment control” (A, C) or cisplatin (B, D), in which the same corresponding concentrations were used (i.e., first dose of the nanocomposites was compared to the first dose of cisplatin).

A) MCF7 — Staistical Analysis compared to the “no treatment” control

Treatment Group	Concentration							
	0.025 mg/ml		0.05 mg/ml		0.1 mg/ml		0.5 mg/ml	
	Significant	P value	Significant	P value	Significant	P value	Significant	P value
**ZIF-8/Silica**	No	0.2338	No	0.3416	No	0.183	Yes	0.0001
**ZIF-8/Silica/Cp**	No	0.0579	No	0.093	Yes	0.0427	Yes	0.0001
**ZIF-8/Silica/GPt**	No	0.9076	No	0.273	No	0.2407	Yes	0.0001
**ZIF-8/Silica/Cp/PEG**	No	0.6002	No	0.4905	Yes	0.0208	Yes	0.0001
**ZIF-8/Silica/GPt/PEG**	No	0.3931	No	0.2588	No	0.1531	Yes	0.0001
	0.001125 mg/ml		0.00225 mg/ml		0.0045 mg/ml		0.0225 mg/ml	
**Cp**	Yes	0.0147	Yes	0.0288	No	0.1347	Yes	0.0045
	0.00945 mg/ml		0.0189 mg/ml		0.0378 mg/ml		0.189 mg/ml	
**GPt**	No	0.4415	No	0.1752	No	0.0638	Yes	0.0275

B) MCF7 — Staistical Analysis compared to cisplatin

Treatment Group	Concentration							
	0.025 mg/ml		0.05 mg/ml		0.1 mg/ml		0.5 mg/ml	
	Significant	P value	Significant	P value	Significant	P value	Significant	P value
**ZIF-8/Silica**	No	0.5753	No	0.972	No	0.9975	No	0.7141
**ZIF-8/Silica/Cp**	No	0.7941	No	0.5291	No	0.8909	No	0.7141
**ZIF-8/Silica/GPt**	No	0.6795	No	0.9861	No	>0.9999	No	0.7141
**ZIF-8/Silica/Cp/PEG**	No	0.5764	No	0.594	No	0.9886	No	0.7141
**ZIF-8/Silica/GPt/PEG**	No	0.9967	No	0.9983	No	0.9989	No	0.7141
	0.001125 mg/ml		0.00225 mg/ml		0.0045 mg/ml		0.0225 mg/ml	
**Cp vs. control**	Yes	0.0147	Yes	0.0288	No	0.1346	Yes	0.0045
	0.00945 mg/ml		0.0189 mg/ml		0.0378 mg/ml		0.189 mg/ml	
**GPt**	No	0.457	No	0.3218	No	0.6489	No	0.2982

C) **HFF**
Staistical Analysis compared to the “no treatment” control

Treatment Group	Concentration							
	0.025 mg/ml		0.05 mg/ml		0.1 mg/ml		0.5 mg/ml	
	Significant	P value	Significant	P value	Significant	P value	Significant	P value
**ZIF-8/Silica**	No	0.0852	No	0.1272	No	0.1001	Yes	0.0001
**ZIF-8/Silica/Cp**	No	0.2191	No	0.2222	No	0.1471	Yes	0.0001
**ZIF-8/Silica/GPt**	Yes	0.0313	No	0.2167	No	0.1891	Yes	0.0001
**ZIF-8/Silica/Cp/PEG**	No	0.1547	No	0.1409	Yes	0.0388	Yes	0.0001
**ZIF-8/Silica/GPt/PEG**	Yes	0.026	Yes	0.0335	No	0.1887	Yes	0.0001
	0.001125 mg/ml		0.00225 mg/ml		0.0045 mg/ml		0.0225 mg/ml	
**Cp**	No	0.1292	No	0.0712	Yes	0.005	Yes	0.0001
	0.00945 mg/ml		0.0189 mg/ml		0.0378 mg/ml		0.189 mg/ml	
**GPt**	No	0.2175	No	0.1849	Yes	0.0197	Yes	0.0007

D) **HFF**
Staistical Analysis compared to cisplatin

Treatment Group	Concentration							
	0.025 mg/ml		0.05 mg/ml		0.1 mg/ml		0.5 mg/ml	
	Significant	P value	Significant	P value	Significant	P value	Significant	P value
**ZIF-8/Silica**	No	0.9896	No	0.733	Yes	0.0409	Statistical analysis cannot be performed since the percentage cell viability for Cp at 0.0225 mg/ml is equal to zero.	
**ZIF-8/Silica/Cp**	No	0.8695	No	0.3707	No	0.1029		
**ZIF-8/Silica/GPt**	No	0.7163	No	0.9412	No	0.2063		
**ZIF-8/Silica/Cp/PEG**	No	0.387	No	0.1255	No	0.0514		
**ZIF-8/Silica/GPt/PEG**	No	0.4071	No	0.1623	No	0.124		
	0.001125 mg/ml		0.00225 mg/ml		0.0045 mg/ml			
**Cp vs. control**	No	0.1292	No	0.0712	Yes	0.0049		
	0.00945 mg/ml		0.0189 mg/ml		0.0378 mg/ml			
**GPt**	No	0.2957	No	0.1083	Yes	0.0019		

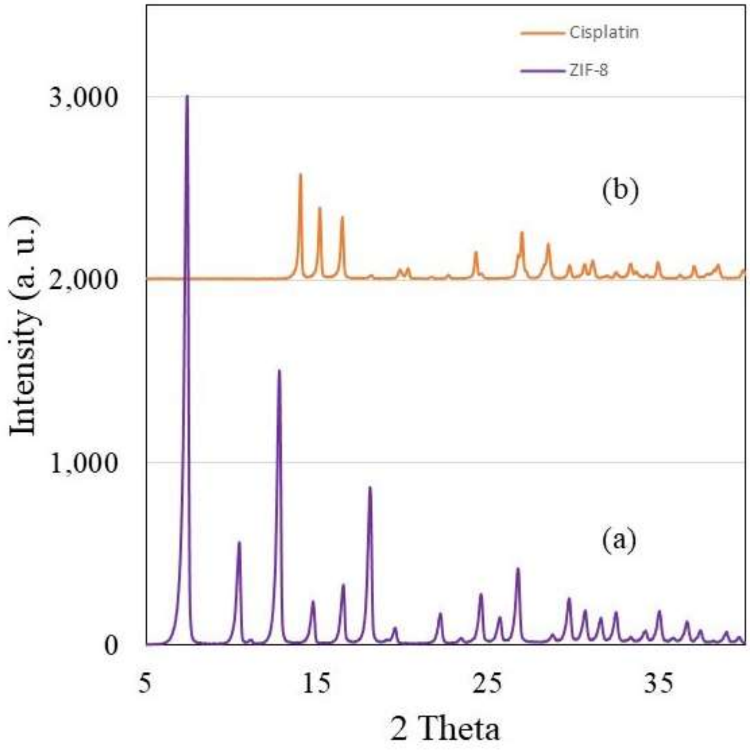

Fig S1. X-ray diffraction pattern of (a) ZIF-8 and (b) cisplatin.

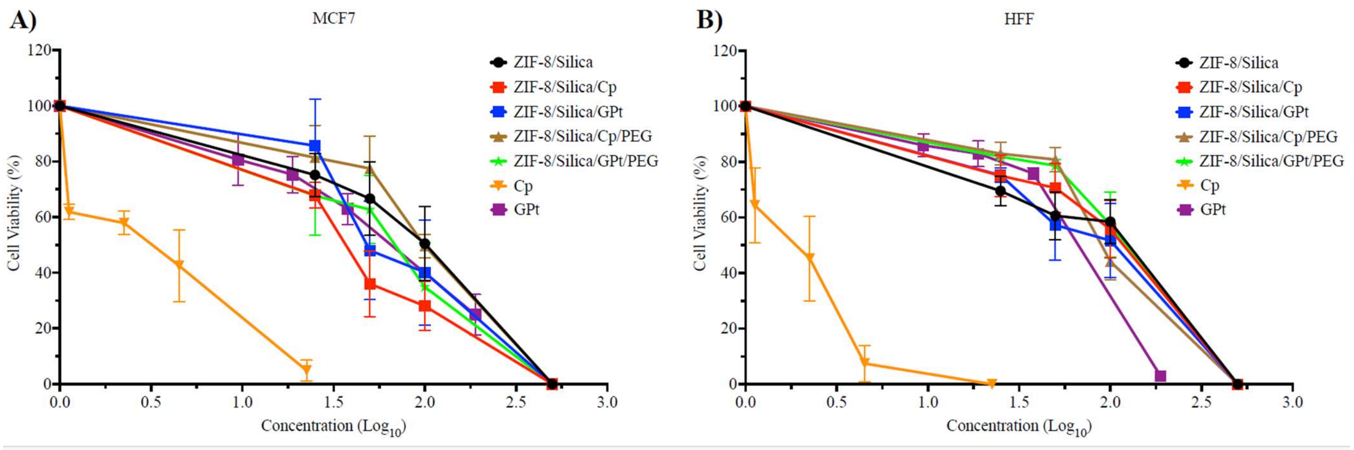

Fig. S2. Data from the cell viability assay presented in a Log scale. A) MCF7 and B) HFF cells were treated with the following conditions: ZIF-8/Silica, ZIF-8/Silica/Cp, ZIF-8/Silica/GPt, ZIF-8/Silica/Cp/PEG, ZIF-8/Silica/GPt/PEG, Cp, and GPt for 48h.
